# Cognitive functioning and clinical characteristics of children with non-syndromic orofacial clefts: A case-control study

**DOI:** 10.3389/fpsyg.2023.1115304

**Published:** 2023-02-28

**Authors:** Kinga Amália Sándor-Bajusz, Tímea Dergez, Edit Molnár, Kinga Hadzsiev, Ágnes Till, Anna Zsigmond, Attila Vástyán, Györgyi Csábi

**Affiliations:** ^1^Division of Child and Adolescent Psychiatry, Department of Pediatrics, Medical School and Clinical Center, University of Pécs, Pécs, Hungary; ^2^Institute of Bioanalysis, Medical School and Clinical Center, University of Pécs, Pécs, Hungary; ^3^Department of Medical Genetics, Medical School and Clinical Center, University of Pécs, Pécs, Hungary; ^4^Division of Pediatric Surgery, Department of Pediatrics, Medical School and Clinical Center, University of Pécs, Pécs, Hungary

**Keywords:** cleft lip, cleft palate, neurodevelopment, executive function, developmental outcomes

## Abstract

**Introduction:**

The higher rate of neuropsychiatric disorders in individuals with non-syndromic orofacial clefts has been well documented by previous studies. Our goal was to identify children with non-syndromic orofacial clefts that are at risk for abnormal neurodevelopment by assessing their developmental history and present cognitive functioning.

**Materials and methods:**

A single-center, case-controlled study was carried out at the Department of Pediatrics of the University of Pécs in Hungary. The study consisted of three phases including questionnaires to collect retrospective clinical data and psychometric tools to assess IQ and executive functioning.

**Results:**

Forty children with non-syndromic oral clefts and 44 age-matched controls participated in the study. Apgar score at 5 min was lower for the cleft group, in addition to delays observed for potty-training and speech development. Psychiatric disorders were more common in the cleft group (15%) than in controls (4.5%), although not statistically significant with small effect size. The cleft group scored lower on the Continuous Performance Test. Subgroup analysis revealed significant associations between higher parental socio-economic status, academic, and cognitive performance in children with non-syndromic orofacial clefts. Analyzes additionally revealed significant associations between early speech and language interventions and higher scores on the Verbal Comprehension Index of the WISC-IV in these children.

**Discussion:**

Children with non-syndromic orofacial clefts seem to be at risk for deficits involving the attention domain of the executive system. These children additionally present with difficulties that affect cognitive and speech development. Children with non-syndromic orofacial clefts show significant skill development and present with similar cognitive strengths as their peers. Longitudinal studies with larger sample sizes are needed to provide more conclusive evidence on cognitive deficits in children with non-syndromic orofacial clefts at risk for neurodevelopmental difficulties.

## 1. Introduction

Orofacial clefts are the most common craniofacial anomalies that affect the lip, palate and/or both structures (Harila et al., 2013; Li et al., 2019). Approximately 30% of oral clefts are associated with a known genetic syndrome (syndromic clefts), however, the remaining 70% occur without a known identified syndrome (non-syndromic clefts; Mossey and Modell, 2012; Saleem et al., 2019). Orofacial clefts (OFCs) are divided into three different subtypes on an anatomically basis; cleft lip (CL), cleft lip and palate (CLP) and cleft palate only (CPO; Lithovius et al., 2014). The higher risk of mental disorders in individuals born with non-syndromic OFCs is well documented in the literature (Richman and Ryan, 2003; Nopoulos et al., 2005, 2010; Boes et al., 2007; Richman et al., 2012; Pedersen et al., 2016; Tillman et al., 2018; Gallagher and Collett, 2019). These children are disproportionately afflicted by psychiatric disorders including schizophrenia, intellectual disability, autism spectrum disorder, anxiety disorders and ADHD (Pedersen et al., 2016; Ansen-Wilson et al., 2018; Tillman et al., 2018). Children with non-syndromic OFCs are also at high risk for learning disabilities (Richman and Ryan, 2003; Tillman et al., 2018; Gallagher and Collett, 2019). Multiple stress factors including repetitive cleft repair surgeries, aesthetics, and functional consequences such as speech difficulty were believed to be the basis of such deficits (Gallagher and Collett, 2019). However, the underlying mechanisms for these deficits have not been clarified (Yang et al., 2012; Gallagher and Collett, 2019). A unified maldevelopment of the brain and facial structures is a possible etiology behind the observed neuropsychiatric disorders in this patient population (Speltz, 2000; Nopoulos et al., 2005; Boes et al., 2007; Weinberg et al., 2009; Yang et al., 2012; Adamson et al., 2014; Ansen-Wilson et al., 2018; Gallagher and Collett, 2019).

Executive dysfunction occurs when cognitive skills responsible for organizing and self-regulating behaviors are impaired (Shaheen, 2014; Zelazo, 2015). Executive functions are interconnected with the maturation of the prefrontal cortex, and their dysfunctions are common in neurodevelopmental and psychiatric disorders (Shaheen, 2014; Zelazo, 2015; Bausela-Herreras et al., 2019; Faedda et al., 2019). Specific patterns of executive dysfunction manifest according to different types of neurodevelopmental disorder and may even be a precursor before the diagnosis of these conditions (Zelazo, 2015; Bausela-Herreras et al., 2019; Otterman et al., 2019). Neuroimaging studies and the underlying cognitive deficits suggest that frontal and prefrontal cortical function may be impaired in children with non-syndromic OFCs (Nopoulos et al., 2010; Adamson et al., 2014; Chollet et al., 2014), and recommend further examination of executive functioning during follow-up (Tillman et al., 2018). Previous studies have examined the executive system in children with non-syndromic OFCs (Nopoulos et al., 2002; Laasonen et al., 2004; Conrad et al., 2009; Lemos and Feniman, 2010; Bodoni et al., 2021), but screened only one or two of its dimensions. It is often unclear whether syndromic participants were excluded from these studies (Gallagher and Collett, 2019), and may include a mixed population of both syndromic and non-syndromic forms (Nopoulos et al., 2000, 2002). Underlying genetic abnormalities—which are present in syndromic oral clefts—often affect proper brain development and function (McDonald-McGinn et al., 2015; Berg et al., 2016) and may therefore misrepresent the non-syndromic population (Rincic et al., 2016; Sándor-Bajusz et al., 2022).

The primary goal of our study was to screen cognitive deficits in children with non-syndromic OFCs to identify an at-risk subpopulation for neurodevelopmental disorders. We further aimed to identify risk factors that may additionally affect the overall neurodevelopmental course of these children. We hypothesized that children with non-syndromic OFCs would present with more cognitive difficulties compared to their non-cleft peers.

## 2. Materials and methods

### 2.1. Design

A single-center, case-controlled study was carried out at the Department of Pediatrics of the University of Pécs in Hungary. The study was approved by the Regional Ethics Committee of the University of Pécs (approval number: 7967-PTE 2020) and was performed in line with the principles of the Declaration of Helsinki. Permission to utilize the materials in the study was granted by the copyright holders (PsyWay, 2020).

### 2.2. Participants

All participating children with non-syndromic OFCs (further mentioned as the cleft group) are patients of the Cleft Team of the Pediatric Surgery Unit, Department of Pediatrics of the University of Pécs. The inclusion criteria consisted of the following: children with non-syndromic OFCs, 6–16 years old and an IQ ≥ 70. An OFC was considered non-syndromic when the cleft was the only single malformation without additional physical or developmental anomalies (Bjørnland et al., 2021). Controls were recruited from the community of Baranya County, specifically from public elementary, high schools, and post advertisements on social media. The inclusion criteria of the controls included the following: healthy children born without oral clefts, 6–16 years old and IQ ≥ 70. Medical geneticists examined all participants of the cleft group to rule out the presence of additional congenital malformations and/or underlying syndromes. The study was carried out between July 2020 and March 2022 in the Department of Pediatrics of the University of Pécs, Hungary. Informed consent was obtained from the parents and participants in the study.

### 2.3. Materials

Initially all psychometric tests were completed on site. Due to the ongoing COVID-19 pandemic, parts of the study were completed online; this included the questionnaires and the four cognitive tests (Stroop, TOL, CPT, and Corsi). Measurements that required in-person completion (IQ test) were postponed onto a later period once the pandemic situation improved.

#### 2.3.1. Questionnaires

A parental questionnaire was developed for the study to collect demographic data. This included prenatal and postnatal history, birth, motor and language development, education, previous psychiatric treatment, and history of somatic and neuropsychiatric disorders. Parental socio-economic data were additionally collected, including parental age, education, and employment status. Parents were also asked regarding family history of neuropsychiatric disorders and/or any previous psychiatric treatment. The Hungarian version of the Child Behavior Checklist (CBCL) was used to screen for behavioral and emotional problems in children and adolescents during the previous 6 months (Achenbach, 1991; Rózsa et al., 1999).

#### 2.3.2. Computer-based cognitive tests

Four computer-based tests were used to assess the main domains of executive functioning. All tests were provided by the Psyway Hungarian psychometric website and all tests are standardized and norm-referenced (PsyWay, 2020). Each cognitive test is summarized in Table 1.

**Table 1:** Cognitive tests used in the study to measure executive functioning.

Cognitive test	EF domain(s) measured	Main outcome measures used in the study
Stroop test	Cognitive flexibility (Diamond, 2013; Parris, 2014; Scarpina and Tagini, 2017)	Inhibition of cognitive interference: speed and accuracy of the response
Tower of London	Planning ability and working memory (Bull et al., 2004; Unterrainer et al., 2004; Kaller et al., 2011; Naidoo et al., 2019)	Total correctly solved trials, total rule violation, mean execution time, average number of trials and weighted performance score
Corsi block-tapping test	Visuo-spatial working memory (Kessels et al., 2000; Brunetti et al., 2014)	Block-span
Continuous performance task	Attention (Conners, 2014; Roebuck et al., 2016)	Detectability (%), omissions (%) and commissions (%)

#### 2.3.3. Intelligence test: WISC-IV (Wechsler intelligence scale for children—Fourth edition)

We used the official Hungarian version of the WISC-IV (Nagyné Réz et al., 2007) to measure full-scale IQ, important for the assessment of executive functioning (Grizzle, 2011; Ardila, 2018).

### 2.4. Procedure

**Figure 1:**
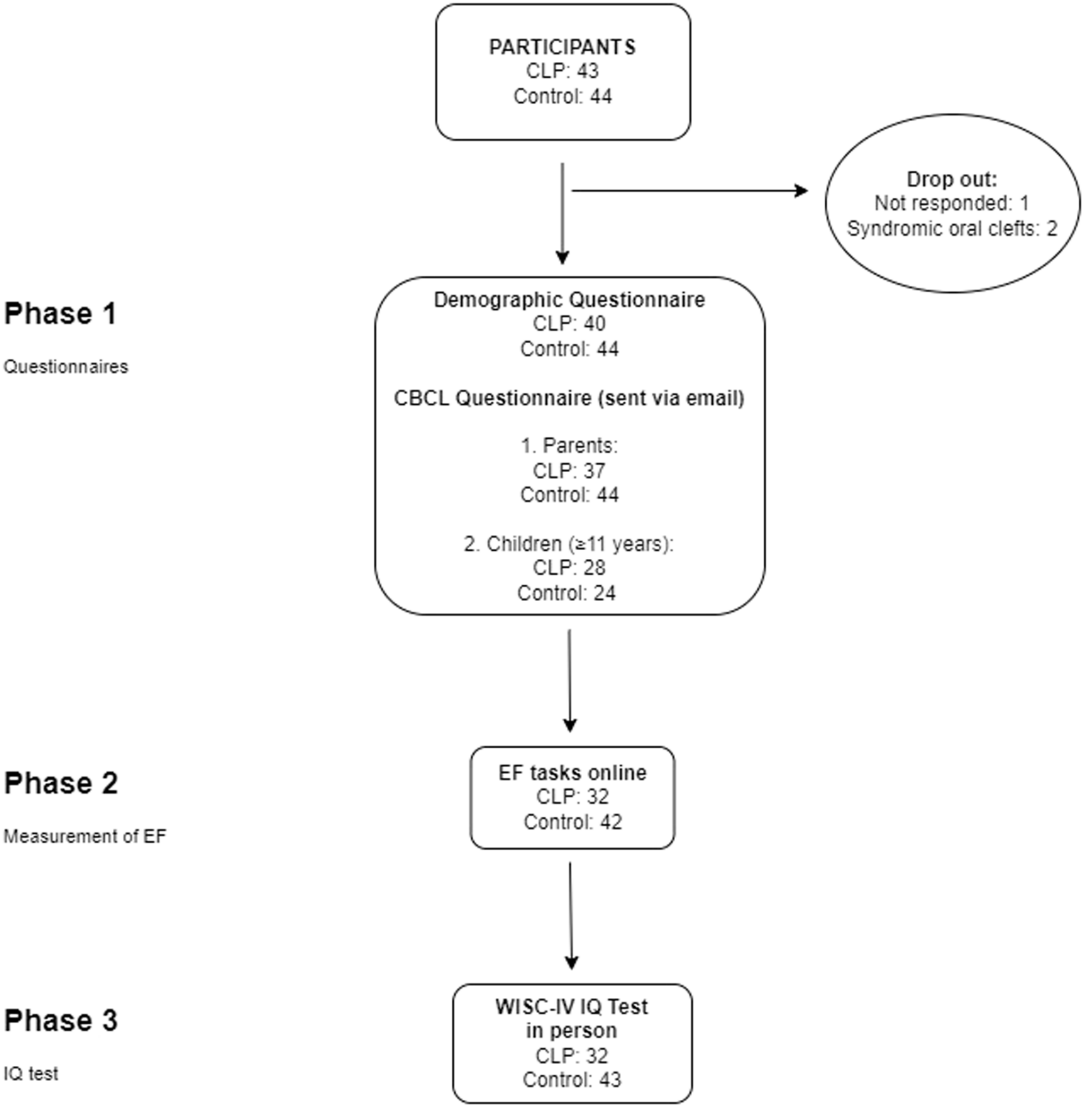
Study flow. The analyzes were divided into three phases. The number of the participants are provided for each phase (CLP, cleft lip and/or palate group; EF, executive function; IQ, intelligence quotient).

The study was divided into three phases, which begun by completing two online questionnaires (Phase 1) followed by online cognitive tasks (Phase 2) and an in-person IQ test (Phase 3, see Figure 1).

### 2.5. Statistics

Statistical analysis was carried out using IBM SPSS Statistics 28 Software. A descriptive statistical analysis was performed. The primary aim of the analysis was to compare the differences in the results of cognitive tests (London Tower, Stroop, Corsi, and Continuous Performance Test), IQ (WISC-IV), CBCL (Child Behavior Checklist) and the demographic parameters between the two study groups. Occupational statuses of the parents were classified as follows: employed, not employed, or retired. Academic levels of the parents were initially grouped into basic (elementary, lower secondary education), intermediate (upper secondary) and advanced (college or university). We later grouped these levels as either higher education (upper secondary education, college, or university) or lower education (elementary, lower secondary education) to increase statistical power.

The raw score is an untransformed score from a measurement of the above listed cognitive tests and the CBCL questionnaire. The raw scores were converted into a scale called *T*-score scale, which assumes a normal distribution with the mean = 50 and the standard deviation = 10. The *T*-scores of all psychometric tests were expressed as means ± standard deviations. The categorical data of the cleft and control groups were analyzed using contingency tables and the chi-squared or Fischer’s test, as appropriate. For quantitative variables, two-sided independent samples Student’s *t*-test were used. The Welch test was applied in cases when the variance was not homogenous. Analysis of variance (ANOVA) was used to test the difference among more than two groups (e.g., in case of analysis based on the type of cleft). These variables follow a normal distribution. Statistical significance was established as a value of *p* of <0.05. Effect sizes were defined as Cohen’s *d* value in case of two independent groups, *η*^2^ in case of ANOVA test, and *ϕ* value in case of Chi-square test (Coe, 2002).

## 3. Results

### 3.1. Participants

We recruited 43 children with non-syndromic OFCsand 44 controls for the study. Past medical history revealed two syndromic OFCs and these participants were excluded from the study. One participant of the cleft group was lost to follow up. The data of 84 study participants were analyzed (see Figure 1).

### 3.2. Cognitive functioning

The CPT revealed differences between the two groups: the cleft group scored lower on detectability (%) than controls (*p =* 0.022, *d =* 0.55, see Table 2). They also missed more targets than controls (*p =* 0.058, *d =* 0.46, see Table 2). We did not observed differences for the remaining cognitive test results (see Supplementary Tables 1–3). None of the participants scored below average in any of the dimensions of the WISC-IV, however controls scored higher on the PRI and WMI subtests (see Supplementary Table 4).

**Table 2:** Results of the CPT (continuous performance task).

Performance measures	Group	*n*	Mean ± SD	*p*-Value	Cohen’s *d*
Detectability (%)	Control	41	59.46 ± 14.90	0.022*	0.55
	Cleft	32	51.03 ± 15.66		
Omission errors (%) (missed targets)	Control	41	59.54 ± 13.00	0.058	0.46
	Cleft	32	53.84 ± 11.84		
Commission errors (%) (false response without target)	Control	41	52.00 ± 12.21	0.47	0.17
	Cleft	32	54.28 ± 14.49		

*Statistical significance. Data are presented as means and standard deviations (SD).

### 3.3. Questionnaires

#### 3.3.1. CBCL questionnaire

##### 3.3.1.1. Children (self-report)

Two dimensions of the CBCL showed significant differences between the groups: controls reported higher symptoms of externalization, somatic, attention, oppositional, and behavioral problems than clefts. Clefts reported higher symptoms of affective problems (see Table 3).

**Table 3:** Results of the CBCL self-report.

Scales	Group	*n*	Mean ± SD	*p*-Value	Cohen’s *d*
Internalization	Control	28	52.57 ± 10.57	0.64	0.13
	Cleft	24	54.17 ± 14.00		
Externalization	Control	28	53.29 ± 8.68	0.024*	0.65
	Cleft	24	47.83 ± 8.05		
Affective problems	Control	28	50.39 ± 8.42	0.39	0.24
	Cleft	24	53.08 ± 13.10		
Anxiety	Control	28	49.50 ± 10.16	0.69	0.11
	Cleft	24	50.71 ± 11.75		
Somatic problems	Control	28	51.60 ± 11.54	0.46	0.21
	Cleft	24	49.42 ± 9.37		
Attention deficit/hyperactivity	Control	28	54.89 ± 10.83	0.24	0.33
	Cleft	24	51.67 ± 8.29		
Oppositional defiance	Control	28	54.25 ± 10.60	0.048*	0.56
	Cleft	24	48.13 ± 11.15		
Behavioral problems	Control	28	51.32 ± 7.61	0.19	0.37
	Cleft	24	48.46 ± 7.90		

*Statistical significance. Data are presented as means and standard deviations (SD).

##### 3.3.1.2. Parental report

Parents of the controls reported higher symptoms across all scales of the CBCL compared to parents of the cleft group, with small effect sizes (see Supplementary Table 5).

#### 3.3.2. Demographic measures

##### 3.3.2.1. Children

###### 3.3.2.1.1. Cleft status

There were no significant differences between the age of cleft versus controls (see Table 4). More than half of the cleft group was represented by boys (56.6%), while controls had more girl participants (67.7%, *p* = 0.031, *ϕ = 0.24*). Three subtypes of OFCs were present in the cleft group: 45% with cleft lip and palate (CLP), 37.5% with cleft lip (CL) and 17.5% with cleft palate only (CPO). Left-sided (32.5%) and bilateral (32.5%) OFCs were the most common. Overall, 29.16% of the cleft group reported their repaired OFCs as a current medical condition. All participants of the cleft group had repaired clefts, and none of these children had persistent hearing deficiency.

**Table 4:** Demographic data of the study groups.

Variable	Cleft group (mean ± SD)	*n*	Control group (mean ± SD)	*n*	*p*-Value	Cohen’s *d*
Age	12.00 ± 2.62	39	11.77 ± 2.63	44	0.69	0.09
Education						
Academic year	6.17 ± 2.38	39	6.06 ± 2.75	44	0.99	0.04
Overall academic score	4.45 ± 0.51	38	4.46 ± 0.58	43	0.95	0.02
Birth						
Week of delivery	38.97 ± 2.19	39	39.20 ± 1.62	44	0.59	0.12
APGAR score 1	8.88 ± 0.62	36	8.97 ± 0.52	41	0.58	0.16
APGAR score 2	9.77 ± 0.59	36	9.97 ± 0.15	41	0.031*	0.48
Birth weight (g)	3414.87 ± 614.58	39	3488.31 ± 618.23	44	0.59	0.12
Birth height (cm)	51.76 ± 4.08	38	50.43 ± 3.32	44	0.11	0.36
Head circumference (cm)	34.75 ± 1.51	16	34.43 ± 1.90	30	0.57	0.19
Motor development						
Rolls over (months)	3.97 ± 0.93	39	4.17 ± 1.02	40	0.37	0.20
Sits (months)	6.50 ± 1.55	38	7.29 ± 2.00	41	0.06	0.44
Crawls (months)	8.61 ± 1.74	38	8.47 ± 1.80	41	0.73	0.08
Walks (months)	11.88 ± 1.38	39	12.02 ± 1.64	43	0.68	0.09
Potty-trained (years)	2.71 ± 0.84	39	2.34 ± 0.54	42	0.008*	0.53
Language development						
First words (months)	15.00 ± 7.65	39	13.50 ± 4.83	37	0.53	0.23
Two-word phrases (months)	24.43 ± 9.77	38	19.52 ± 6.11	34	0.039*	0.60
Coherent sentences (year)	2.50 ± 0.75	38	2.22 ± 0.59	38	0.055	0.41
Parental SES						
Gravidity of mother	2.44 ± 1.37	39	2.66 ± 1.94	44	0.99	0.13
Mother’s age	42.79 ± 4.43	39	44.67 ± 4.57	43	0.063	0.42
Father’s age	45.71 ± 5.06	39	48.13 ± 5.24	43	0.037*	0.47

Data are presented as means and standard deviations (SD). The number of participants is provided for each variable (*n*). Units are provided for each measurement. Overall academic score was provided according to the 5-point grade system used in Hungary, which defines 1 as insufficient, 2 as sufficient, 3 as satisfactory, 4 as good and 5 as excellent.

###### 3.3.2.1.2. Academic performance and past psychiatric history

We observed no differences in the overall academic score; both clefts and controls achieved a good overall score in the current academic year (see Table 4). Preschool integration was significantly more difficult for the cleft group compared to controls (*p* = 0.025*, ϕ =* 0.26). Both study groups did well later in preschool without requiring grade repetition (*p* = 0.96*, ϕ =* 0.005). Children of the cleft group were examined by pedagogical professional services more often than controls (*p* < 0.001*, ϕ =* 0.49). Participants in the cleft group required special education plans more often than controls (*p* = 0.016*, ϕ =* 0.29). There were no differences in the rate of elementary grade repetition between clefts and controls (*p* = 0.60*, ϕ =* 0.073). We observed a higher proportion of psychiatric disorders in the cleft group (15%) compared to controls (4.5%; *p* = 0.14*, ϕ =* 0.18). The cleft group received previous psychiatric therapy more often (15%) than controls (0%; *p* = 0.009*, ϕ =* 0.29). The reported psychiatric diagnoses were ADHD (50%), borderline personality disorder (12.5%), learning disability (12.5%), depression (12.5%) and anxiety disorder (12.5%). Children in the cleft group required additional support for learning, psychological and physical well-being during their education more often than controls (*p* < 0.001*, ϕ =* 0.49), specifically speech and language therapy (*p* < 0.001*, ϕ =* 0.51). Overall, 4.5% of controls reported having a psychiatric comorbidity, which included dyslexia (50%) and ADHD (50%).

###### 3.3.2.1.3. Pregnancy and developmental history

All participating children were born full-term *via* uncomplicated births. No differences were observed in the total number of pregnancies, and natural and caesarian delivery (*p* = 0.63*, ϕ =* 0.05). Apgar score at 5 min was lower in the cleft group (*p =* 0.031, *d =* 0.48, see Table 4). No differences were observed in the week of delivery, head circumference and birthweight between the two study groups (see Table 4). The need for postnatal supportive care did not differ between clefts and controls (respiratory support, surfactant therapy, phototherapy, antibiotics, and transfusions; *p* = 0.23, *ϕ =* 0.13). Mothers of the cleft group reported feeding (*p* = 0.007, *ϕ =* 0.29) and hearing (*p* < 0.001, *ϕ =* 0.51) difficulties more often than mothers of controls. The cleft group developed motor skills (roll over, sitting) later than controls, however the effect sizes were small (see Table 4). The cleft group was potty trained at an older age than controls (*p =* 0.008, *d =* 0.53, see Table 4). Parents of the cleft group reported that their children were able to form two-word sentences at a later age compared to reports of parents of controls (*p =* 0.039, *d =* 0,60, see Table 4). First words and coherent sentences were also spoken later by children in the cleft group (See Table 4).

##### 3.3.2.2. Parents

###### 3.3.2.2.1. Age, marital and employment status

Parents of the control group were older at the time of assessment than those of the cleft group (see Table 4). Mothers of the cleft group gave birth to their child at an older age than mothers of controls (*p* = 0.50, *d =* 0.05). Most parents of clefts (70.0%) and controls (69.8%) were married, and no differences were observed between the relationship statuses of parents of both groups (*p =* 0.47, *ϕ = 0.08*). The employment statuses of fathers (*p =* 0.42, *ϕ =* 0.25) and mothers (*p* = 0.86, *ϕ =* 0.19*)* did not differ between the two groups.

###### 3.3.2.2.2. Past psychiatric and academic history

History of psychiatric disorders was more often reported by parents of controls (27.3%) compared to clefts (7.5%; *p* = 0.010*, ϕ =* 0.39). One parent of the control group reported to have history of anxiety, but most parents did not further specify these conditions. The majority of reported psychiatric diagnoses in the family of the cleft group were depression (75%) or anxiety disorders (25%). Most parents completed high school and/or had a university degree. Significant differences were not observed in the mother’s level of education between the two study groups (*p =* 0.29, *ϕ =* 0.12). Fathers of the control group achieved a higher degree of education than fathers of the cleft group who had lower secondary education (*p =* 0.024, *ϕ = 0.25*).

### 3.4. Subgroup analysis of the cleft group

Following data collection and analyzes, we hypothesized that the more complex cleft subtypes would obtain lower scores on the IQ test, and present with a history of atypical neurodevelopment, academic difficulties, and psychiatric disorders. We further assumed that early interventions for speech and language would positively impact cognitive development, and the later would be reflected in the IQ score of these children.

A total of 10 girls and 30 boys were tested in the cleft group (see Table 5): Boys became potty-trained earlier (2.39 years) than girls (3.50 years; *p* = 0.037*, d =* 0.79). Hearing difficulties were in highest proportion for CPO (57.1%) than for CL (13.3%) and CPL (44.4%) however with small effect size (*p* = 0 0.063, *d =* 0.36). In the analysis according to types of clefts, CLP was the subtype that was most often referred to special education services: CL in 40%, CPO in 14% and CLP in 72% of the cases (*p =* 0.023, *d =* 0.29). CLP subtype was diagnosed with psychiatric comorbidities in highest proportion (22.2%) compared to CL (13.3%) and CPO (0%) (*p =* 0.53, *d =* 0.22). CLP subtype had additionally received previous psychiatric care in highest proportion (22.2%) compared to the rest of the cleft subtypes (*p =* 0.61*, d =* 0.23). Left (15.4%) and bilateral (30.8%) sided clefts presented the highest proportion of psychiatric diagnoses (*p =* 0.27*, d =* 0.35). The relationship between parental socioeconomic status (SES) and children’s cognitive performance.

**Table 5:** Demographical data of the orofacial cleft group.

Variable	*n*
Age	
Younger group (6–11 years)	18
Older group (12–16 years)	22
Sex	
Male	30
Female	10
Type of orofacial cleft	
CLP	18
CPO	7
CL	15
Side of orofacial cleft	
Right	8
Left	13
Bilateral	13
Midline	6

CLP, cleft lip and palate; CPO, cleft palate only; CL, cleft lip.

We aimed to explore variables of parental SES that may influence the outcome of academic and cognitive performance. Fathers with a high academic background reached a higher overall academic average compared to children with fathers of low academic background (*p =* 0.005*, d =* 0.79). Children with mothers of a high academic background reached a higher overall academic average compared to children with mothers of a low academic background (see Table 6). The same pattern was observed for the IQ scores: children who scored higher on almost all indexes of the IQ had parents with a higher academic background (see Supplementary Tables 6, 7). A total of 44.4% of cleft children with single parents had a psychiatric condition(s), while only 6.5% had psychiatric condition(s) when raised by married parents (*p =* 0.016*, d =* 0.44).

**Table 6:** Parental level of education in relation to overall academic average of the cleft group.

Level of education		*n*	Mean ± SD	*p*-Value	Cohen’s *d*
Father	High	25	4.60 ± 0.42	0.005*	1,02
	Low	14	4.11 ± 0.57		
Mother	High	29	4.62 ± 0.42	<0.001*	1.88
	Low	10	3.85 ± 0.38		

*Statistical significance.

#### 3.4.1. The relationship between speech/language therapy and the IQ score

We explored the effect of speech and language therapy on IQ scores and overall academic average. FS-IQ and VCI scores were higher for children who received therapy (see Table 7). Overall academic average was higher for cleft participants who did not undergo therapy, although with small effect size (see Table 7). A one-way ANOVA was performed to compare the effect of the affected side of the cleft (left, right, bilateral and midline) on IQ scores. We observed differences for continuous variables in WMl when tested by the affected side (*p =* 0.037, *η^2^ =* 0.27, see Supplementary Table 8).

**Table 7:** Effect of speech and language therapy on IQ scores and overall academic average.

Cognitive performance	Speech and language therapy	*n*	Mean ± SD	*p*-Value	Cohen’s *d*
FS-IQ	No	16	107.06 ± 10.77	0.077	0.66
	Received	15	114.13 ± 10.68		
VCI	No	16	109.44 ± 10.73	0.005*	1.10
	Received	15	121.20 ± 10.63		
PRI	No	16	104.50 ± 10.67	0.24	0.43
	Received	15	108.67 ± 8.44		
WMI	No	16	102.38 ± 13.88	0.55	0.22
	Received	15	105.13 ± 11.54		
PSI	No	16	103.63 ± 9.02	0.83	0.07
	Received	15	104.53 ± 14.22		
Overall academic average	No	18	4.54 ± 0.48	0.22	0.40
	Received	21	4.33 ± 0.56		

FS-IQ, full-scale IQ; VCI, verbal comprehension index; PRI, perceptual reasoning index; WMI, working memory index; PSI, processing speed index.

## 4. Discussion

We analyzed the cognitive functioning and clinical characteristics of 40 children with non-syndromic OFCs and 44 age-matched controls. All participants performed well on the executive function tasks, except for the CPT; children with non-syndromic OFCs scored lower and missed targets more often than controls (omission errors, see Table 4). The results raise the possibility of an underlying attention deficit in these children described previously by other studies (Nopoulos et al., 2010; Pedersen et al., 2016). The two groups scored within normal ranges on the IQ test, however controls scored higher on the PRI and WMI subtests. Subgroup analysis of the cleft group revealed significant relationships between parental SES and IQ scores: children of parents with a higher educational background scored significantly higher on the IQ test, specifically reflected in perceptual reasoning and the full-scare IQ score. We also observed a significant association between early intervention and IQ: children who received speech and language therapy achieved higher scores specifically reflected in the verbal component (VCI) of the WISC-IV (see Table 7). We further observed the influence of family structure on mental health outcomes: children raised by single parents were diagnosed with psychiatric conditions more often than children raised by married parents.

Children of the control group reported more symptoms of externalizing disorders (attention, oppositional, behavioral), while children with non-syndromic OFCs reported symptoms of internalizing disorders (affective, anxiety) more than controls (Table 3). Parents of the control group reported higher symptoms across all scales of the CBCL. However, retrospective analysis of past medical history revealed that children with non-syndromic OFCs were clinically diagnosed with psychiatric disorders at a higher proportion and received psychiatric support more often than controls. Larger cohort studies have previously described this observation (Pedersen et al., 2016; Tillman et al., 2018). While there is a clear difference in the proportion of psychiatric disorders between our two study groups, this is not statistically detectable, and the effect size is small. A larger sample may provide conclusive evidence of this observation.

Psychiatric diagnoses varied across cleft subtypes and the affected side: the highest proportion of psychiatric diagnoses were observed in CLP, and bilateral-sided clefts. These observations may suggest that the more complicated clefts more likely present with psychiatric comorbidities (Pedersen et al., 2016; Gallagher et al., 2018). We did not observe psychiatric comorbidities in CPO children, which is in contrast with previous observations (Nilsson et al., 2015; Pedersen et al., 2016; Tillman et al., 2018; Gallagher and Collett, 2019). Interestingly, less than half (29.16%) of the cleft group participants recognized their repaired OFC as a disease or medical condition. This may indicate that the causative stressor is in fact something other than the physical awareness of the defect itself (Aleksieva et al., 2021). Apgar score at 5 min was lower for the cleft group than for controls, but clinically within the normal range. We observed no further complications in the postnatal period between the two study groups. There was a tendency of a slower onset of developmental milestones in children with OFCs; potty-training and the use of two-word phrases presented at a later age compared to controls, also within clinical ranges. Children with OFCs experienced difficulties integrating into preschool, and most required additional support for learning, psychological and physical well-being throughout their education. Difficulties with speech and language development are known to be a consequence related to the primary defect; however, studies highlight the possibility of a central auditory dysfunction, which may cause developmental issues that affect these skills (Čeponien et al., 1999; Yang et al., 2012; Conrad et al., 2021). Based on our results, children with non-syndromic OFCs initially have a slower development and experience difficulties integrating into preschool; however, it seems that they go through a “catch-up phase” around school age and perform well—almost equal to their peers—throughout elementary and high school.

Our study has important limitations. The small sample size of the study, limited us to further explore relationships within gender, cleft subtype and affected side. The sample size varied across the different phases of the study. Most of the children in the cleft group were represented by males. The retrospective nature of the questionnaires may have created bias in the data provided. We could not assess the baseline level of executive functioning prior to the interventional programs (speech and language therapy), and we may observe an overall “corrected” level of cognitive functioning. However, this study has several strengths. Our study is the first to provide data on cognitive performance and clinical characteristics of Hungarian children with non-syndromic OFCs across a wide age-range. We were able to provide data on neurodevelopmental differences in children with non-syndromic OFCs in early infancy and the preschool period. We further demonstrated how these children, despite having previous difficulties during early infancy, can “catch-up” to their peers and perform well. Early intervention, additional help in school and proper parental support seem to have a strong effect on proper cognitive development for this patient population. Our observations suggest the presence of attention deficit in children with non-syndromic OFCs in support of the higher proportion of ADHD diagnosis seen in this population compared to controls. Assessing the executive system at an earlier stage of development, prior to interventional programs, may be useful to screen and identify individuals within the cleft population who are at risk for atypical neurodevelopment.

Children with non-syndromic OFCs seem to be at risk for atypical cognitive and speech development. This may be explained by a unified brain and facial maldevelopment *in utero*. Future studies with large sample sizes are needed to further explore this underlying etiology to identify this subpopulation, since not all children with non-syndromic OFCs present with such difficulties. Longitudinal studies are needed to provide more evidence of baseline cognitive functioning to study early signs of atypical neurodevelopment and the effect of early interventions. Under the right environment, these children present with similar cognitive strengths as their peers and show significant skill development. A good multidisciplinary team, early interventions, special education programs, and proper parental support allow most children with non-syndromic OFCs to perform just as well as other children.

## Data availability statement

The raw data supporting the conclusions of this article will be made available by the authors, without undue reservation.

## Ethics statement

The studies involving human participants were reviewed and approved by Regional Ethics Committee of the University of Pécs (approval number: 7967-PTE 2020). Written informed consent to participate in this study was provided by the participants’ legal guardian/next of kin.

## Author contributions

KS-B, GC, AV, and KH contributed to conception and design of the study. KS-B, ÁT, AZ, KH, EM, and AV collected the data and organized the database. GC supervised the study. TD performed the statistical analysis. KS-B wrote the first draft of the manuscript. GC and TD wrote sections of the manuscript. All authors contributed to the article and approved the submitted version.

## Funding

The authors received financial support from the University of Pécs for the open-access publication of this manuscript.

## Conflict of interest

The authors declare that the research was conducted in the absence of any commercial or financial relationships that could be construed as a potential conflict of interest.

## Publisher’s note

All claims expressed in this article are solely those of the authors and do not necessarily represent those of their affiliated organizations, or those of the publisher, the editors and the reviewers. Any product that may be evaluated in this article, or claim that may be made by its manufacturer, is not guaranteed or endorsed by the publisher.
