# Supplementary material for: Cognitive functioning and clinical characteristics of children with non-syndromic orofacial clefts: A case-control study
Source: Front Psychol. 2023 Feb 28;14:1115304. doi: 10.3389/fpsyg.2023.1115304 (PMC10011643; doi:10.3389/fpsyg.2023.1115304)
Supplement: Supplementary file 1 [file Data_Sheet_1.pdf]

Supplementary Material

Cognitive functioning and clinical characteristics of children with non-syndromic orofacial clefts: A case-control study.

Kinga Amália Sándor-Bajusz*, Tímea Dergez, Edit Molnár, Kinga Hadzsiev, Ágnes Till, Anna Zsigmond, Attila Vástyán, Györgyi Csábi

* Correspondence: Kinga Amália Sándor-Bajusz, sandor.kinga@pte.hu

Supplementary Tables

Supplementary Table 1. Results of the Stroop Test. Data are provided in means (M) and standard deviations (SD).

**Interference**	***Group***	***n***	***M±SD***	***p* value**	**Cohen's *d***
**Speed**	control	42	48.93±6.66	0.48	0.16
	cleft	32	47.67±8.59		
**Accuracy**	control	42	46.21±14.63	0.28	0.26
	cleft	32	49.72±12.52		

Supplementary Table 2. Results of the TOL (Tower of London Task). Data are provided in means and standard deviations (SD).

**Performance measures**	**Group**	***n***	***M±SD***	***p* value**	**Cohen's** d
**Total correctly solved trials**	control	40	49.03±11.88	0.70	0.09
	cleft	31	47.81±14.84		
**Total rule violation**	control	40	49.03±11.88	0.77	0.07
	cleft	31	49.90±12.88		
**Mean execution time**	control	40	37.53±15.84	0.97	0.01
	cleft	31	37.35±16.41		

**Average number of trials**	control	40	41.18±14.68	0.51	0.16
	cleft	31	43.48±14.38		
**Weighted performance score**	control	40	54.93±11.73	0.83	0.05
	cleft	31	54.32±11.18		

Supplementary Table 3. Corsi Block Span Test. Data are provided in means (M) and standard deviations (SD).

**Performance measure**	**Group**	***n***	***M±SD***	***p* value**	**Cohen's *d***
**Block Span**	control	42	53.67±11.39	0.50	0.16
	cleft	32	55.38±9.60		

Supplementary Table 4. The IQ scores of both study groups. Data are provided in means (M) and standard deviations (SD). All four indexes of the IQ were measured, and a full-scale IQ (FS-IQ) score is provided below (VCI: Verbal Comprehension Index, PRI: Perceptual Reasoning Index, WMI: Working Memory Index, PSI: Processing Speed Index).

	**Group**	***n***	***M±SD***	***p* value**	**Cohen's *d***
**Age**	Control	43	11.60±2.74	0.48	0.17
	Cleft	32	12.03±2.39		
**VCl**	Control	43	116.91±10.75	0.66	0.10
	Cleft	32	115.72±12.35		
**PRI**	Control	43	109.16±12.90	0.35	0.22
	Cleft	32	106.63±9.60		
**WMI**	Control	43	107.12±13.87		

	Cleft	32	103.78±12.47	0.29	0.25
**PSI**	Control	43	102.88±10.00	0.59	0.12
	Cleft	32	104.22±11.47		
**FS-lQ**	Control	43	112.72±12.05	0.49	0.16
	Cleft	32	110.81±11.12		

Supplementary Table 5. Results of the CBCL Parental Report. Data are provided in means (M) and standard deviations (SD).

**Scales**	**Group**	***n***	***M±SD***	***p* value**	**Cohen's *d***
**Internalization**	Control	44	54.15±15.70	0.31	0.23
	Cleft	37	51.51±10.08		
**Externalization**	Control	44	50.18±7.72	0.15	0.32
	Cleft	37	47.49±8.36		
**Affective problems**	Control	44	54.98±14.42	0.35	0.21
	Cleft	37	52.57±10.26		
**Anxiety**	Control	44	51.16±13.44	0.54	0.12
	Cleft	37	49.78±9.56		
**Somatic problems**	Control	44	54.91±14.64	0.74	0.08
	Cleft	37	54.12±13.09		
**Attention deficit/hyperactivity**	Control	44	52.49±12.04	0.31	0.23
	Cleft	37	49.97±8.06		
**Oppositional defiance**	Control	44	51.27±9.82	0.11	0.36
	Cleft	37	47.54±9.51		

**Behavioral problems**	Control	44	49.29±6.79	0.25	0.26
	Cleft	37	47.38±7.62		

Supplementary Table 6. Fathers' level of education in relation to the IQ scores of the cleft group. Data are provided in means (M) and standard deviations (SD). FS-IQ: Full-scale IQ, VCI: Verbal Comprehension Index, PRI: Perceptual Reasoning Index, WMI: Working Memory Index, PSI: Processing Speed Index

**IQ Indexes**	**Fathers' level of education**	***n***	***M±SD***	***p* value**	**Cohen's *d***
**FS-IQ**	Low	11	103.82±9.11	.011*	1.04
	High	20	114.15±10.61		
**VCI**	Low	11	111.36±10.54	0.20	0.50
	High	20	117.20±12.63		
**PRI**	Low	11	101.82±10.33	.044*	0.77
	High	20	109.10±8.57		
**WMI**	Low	11	97.09±10.75	0.028*	0.88
	High	20	107.35±12.38		
**PSI**	Low	11	97.91±9.87	0.026*	0.9
	High	20	107.45±11.33		

Supplementary Table 7. Mothers' level of education in relation to the IQ scores of the cleft group. Data are provided in means (M) and standard deviations (SD). FS-IQ: Full-scale IQ, VCI: Verbal Comprehension Index, PRI: Perceptual Reasoning Index, WMI: Working Memory Index, PSI: Processing Speed Index

**IQ Indexes**	**Mothers' level of education**	***n***	***M±SD***	***p* value**	**Cohen's *d***

**FS-IQ**	Low	7	101.71±6.70	0.015*	1.25
	High	24	113.04±10.96		
**VCI**	Low	7	109.71±9.25	0.18	0.64
	High	24	116.71±12.52		
**PRI**	Low	7	96.29±6.78	0.001*	1.73
	High	24	109.51±8.39		
**WMI**	Low	7	96.29±9.62	0.078	0.85
	High	24	105.88±12.78		
**PSI**	Low	7	101.86±12.67	0.58	0.23
	High level	24	104.70±11.52		

Supplementary Table 8. Results of one-way ANOVA which was performed to compare the effect of the affected side of the cleft on IQ. VCI: Verbal Comprehension Index, PRI: Perceptual Reasoning Index, WMI: Working Memory Index, PSI: Processing Speed Index, FS-IQ: Full-scale IQ.

	n	**Affected side**	***M±SD***	***p***	***η²***
**FS-IQ**	5	right	118.00±12.31	0.34	0.12
	12	left	108.83±9.01		
	10	midline	107.60±12.25		
	4	bilateral	113.25±12.20		
**VCI**	5	right	116.60±14,54	0.85	0.029
	12	left	116.83±9.55		
	10	midline	112.30±14.84		
	4	bilateral	115.25±11.90		
**PRI**	5	right	112.80±7.95	0.33	0.12
	12	left	106.33±8.94		
	10	midline	103.00±11.44		
	4	bilateral	108.00±8.16		
**WMI**	5	right	117.20±10.99	0.037*	0.27
	12	left	98.17±11.24		
	10	midline	103.50±11.11		
	4	bilateral	104.00±13.54		
**PSI**	5	right	108.00±10.02	0.36	0.11
	12	left	100.58±9.65		

10	midline	103.30±12.91
4	bilateral	111.50±14.98
